# Prevalence of cognitive impairment among older adult Palestinians in community and nursing home settings

**DOI:** 10.1371/journal.pone.0356393

**Published:** 2026-08-19

**Authors:** Osamah Azzam, Iyad Nazzal, Usama AlKhuffash, Ahmad Abuhassan, Mohammad Abuawad, Ramzi Shawahna

**Affiliations:** 1 Doctor of Medicine program, Department of Medicine, Faculty of Medicine and Allied Medical Sciences, An-Najah National University, Nablus, Palestine; 2 An-Najah National University Hospital, Department of Neurology, An-Najah National University, Nablus, Palestine; 3 Department of Biomedical Sciences and Basic Clinical Skills, Faculty of Medicine and Allied Medical Sciences, An-Najah National University (www.najah.edu), Nablus, Palestine; 4 Clinical Research Center, An-Najah National University Hospital, Nablus, Palestine; Essen University Medical School, GERMANY

## Abstract

**Background:**

Cognitive impairment in older adults represents a critical global public health challenge. This study aimed to describe the prevalence of cognitive impairment and the clinical characteristics of Palestinian older adults living in nursing homes and in the community.

**Methods:**

A cross-sectional study was conducted from May to October 2024 among 441 Palestinians aged 55 years and older, recruited from 10 charitable nursing homes and primary healthcare centers across the West Bank. Cognitive function was evaluated using the validated Arabic version of the Mini-Mental State Examination A-MMSE (GTD-USJ). Comprehensive sociodemographic, clinical, and lifestyle information was collected to describe the two groups.

**Results:**

Of the 441 participants, 305 were recruited from primary healthcare centers and 136 from nursing homes. The median age of residents in nursing homes was 73.0 years. The population had a high prevalence of illiteracy (41.9%) and was predominantly female (66.9%). Clinically, 58% were obese or overweight. The most common illnesses were hypertension (47.8%) and diabetes (30.9%). On the other hand, the median age of primary health care attendees was 65.0 years. The population was 45.2% male, 27.9% smokers, 16.1% illiterate, and 27.5% had completed university. The most common conditions were dyslipidemia (59%), diabetes (50.2%), and hypertension (67.9%). In the nursing home cohort, significant associations with cognitive impairment were identified for gender (p = 0.030), smoking status (p < 0.001), educational attainment (p < 0.001), and monolingualism (p < 0.001). Among primary healthcare attendees, cognitive impairment was significantly correlated with gender (p < 0.001), smoking status (p < 0.001), educational attainment (p < 0.001), and monolingualism (p = 0.003).

**Conclusion:**

This descriptive study documents the first baseline prevalence data for both care settings and nursing home residents in Palestine and underscores the need for geriatric care standards tailored to the local population. The data support investment in longitudinal cohort research to characterize the natural history of cognitive decline in this population, as well as enhanced management of modifiable risk factors and evidence-based cognitive stimulation programs within nursing facilities.

## Background

Cognitive impairment, which spans a continuum from the normal cognitive changes of aging to overt dementia, represents a critical public health challenge worldwide [1,2]. Mild cognitive impairment (MCI) is often conceptualized as an intermediate state characterized by measurable deficits in cognitive functioning that, although not severe enough to disrupt daily activities, exceed what is typically expected with normal aging [3–5]. Epidemiological studies report a wide range in MCI prevalence—from less than 1% to over 40% in older populations—depending largely on the diagnostic criteria and study settings employed [6]. Furthermore, annual progression rates from MCI to dementia are estimated to range between 8% and 15% [6,7]. Importantly, while many individuals with MCI are at increased risk of cognitive decline, a proportion may remain stable or even recover, underscoring the complexity inherent to subclinical cognitive changes.

Defining and diagnosing MCI remain subjects of considerable debate, primarily due to the absence of universally accepted criteria [8,9]. Early attempts to standardize its definition, such as those proposed by Petersen and colleagues, have been criticized as overly restrictive [6,10]. Broader diagnostic constructs—such as cognitive impairment no dementia (CIND)—often yield higher prevalence rates and may better capture the array of cognitive deficits observed in the community [11–14]. Discrepancies in the cut-off scores used to delineate cognitive deficits have further complicated classification, while the traditional exclusion of individuals with significant comorbidities has raised concerns regarding the generalizability and applicability of existing criteria. These issues have prompted revisions by international working groups and institutions—most notably, the refinements made by the National Institute on Aging and the Alzheimer’s Association (NIA-AA) and the introduction of mild neurocognitive disorder in the DSM-5—which collectively aim to encompass a broader spectrum of cognitive dysfunction [15].

The prevalence of cognitive impairment varies considerably depending on both the specific population under investigation and the diagnostic criteria applied [16–18]. For instance, a recent meta-analysis among community-dwelling adults aged over 50 reported prevalence rates ranging from 5.1% to 41.0% and an incidence ranging from 22.0 to 76.8 per 1000 person-years [19]. A systematic review incorporating 53 studies and a combined sample of 376,039 participants from 17 countries, with mean ages ranging from 64.4 to 86.9 years, reported a pooled prevalence of mild cognitive impairment of 21.2% (95% CI: 18.7–23.6%) among older adults residing in nursing homes [20]. Moreover, a study in China found that 22.2% of community individuals aged 60 or older exhibited signs of cognitive impairment [21], whereas among nursing home residents aged 65 or older, the prevalence was markedly higher at 64.0% [22], with these percentages increasing significantly with age. Additionally, cognitive impairment tends to be more prevalent in females than in males among community dwellers—with rates of 17.5% for females versus 15.2% for males—and this gender disparity is even more pronounced in nursing homes, with prevalence rates of 65.6% for females in comparison to 61.2% for males [22]. Previous research consistently indicates that older adult individuals residing in nursing homes are more susceptible to cognitive impairment than those in the general community; indeed, the true prevalence may exceed the figures reported by nursing home administrators [16–19,22]. This underestimation is particularly concerning given that cognitive impairment, especially when progressing to dementia, is strongly associated with elevated risks of depression, fall-related injuries, malnutrition, and overall mortality [23–25]. Consequently, failure to accurately identify cognitive impairment may lead to significant adverse effects on the health and quality of life of older adults.

In Palestine, research on the epidemiology of cognitive impairment among older adults is limited, and studies rarely compare community-dwelling individuals with those residing in institutional settings. Given the unique socio-economic and cultural dynamics of the region—where factors such as limited healthcare resources, socio-political stressors, educational disparities, and traditional family support structures may significantly influence cognitive health —it is imperative to generate local data to inform tailored intervention strategies. This study aims to describe the prevalence of cognitive impairment and its associated risk factors among older Palestinian adults attending primary healthcare centers and those residing in nursing homes. The research seeks to provide robust epidemiological insights and to contribute to the development of evidence-based screening and preventive measures for cognitive decline in the region.

## Methods

### Study design and setting

This descriptive–analytical, cross-sectional study was conducted among older adults aged 55 years and older in the West Bank of Palestine from May to October 2024. The study described the prevalence and associated risk factors for cognitive impairment among individuals residing in nursing homes and those who regularly attend primary healthcare centers. Most nursing homes in Palestine are considered civil society organizations and are usually funded by charitable organizations. These facilities generally provide basic accommodation, meals, and assistance with activities of daily living, with variable access to on-site medical or rehabilitative services. Nursing homes in Palestine are a distinct group whose admission is typically not by choice. Instead, it is primarily driven by circumstances such as social isolation, lack of family support, functional or cognitive decline, chronic health issues, and financial hardship. Of the 14 nursing homes initially identified in the West Bank, 3 were excluded due to geographic constraints in Qbeibeh, Bethlehem, and Jericho. All 11 of the remaining homes were contacted. One in Nablus declined to participate, providing no specific reason. Therefore, data were collected from 10 charitable nursing homes (4 in Ramallah, 2 in Nablus, 2 in Tulkarm, 1 in Jenin, and 1 in Salfit), as well as from primary healthcare centers in the governorates of Jenin, Tulkarm, Nablus, Salfit, Qalqilya, and Ramallah. It is noteworthy that the predominant culture in Palestinian society is one of caring for older adults. Nursing home culture is not very common, so nursing homes are typically used by socially isolated older adults or by those who require a higher level of care.

### Inclusion and exclusion criteria

The inclusion criteria were as follows: individuals aged 55 years and older who were willing to participate, able to provide all necessary information, and who provided informed consent. The age of 55 was chosen as a minimum instead of the “traditional” 60/65 because of the conflict situation and the proportion of displaced citizens and refugees, which may cause Palestinian adults to “age” faster [26] Additionally, the inclusion criteria for selecting these nursing homes were: the nursing home must be located in the West Bank of Palestine, the home must be accessible for the study purposes, and the home’s administration must agree to participate in the study. Exclusion criteria included individuals not residing in the West Bank; those with congenital anomalies or syndromes; individuals experiencing visual or auditory impairments or active psychiatric symptoms that prevented them from completing the assessment; individuals in a state of coma or aphasia; those at end-of-life status; and respondents with incomplete data. It is important to mention that the widespread blockage of West Bank roads that persists to this day has made it highly difficult to reach governorates that are geographically far from the living quarters of the researchers, namely Bethlehem, Jericho, and Jerusalem

### Sample size and sampling technique

According to a recent meta-analysis among community-dwelling adults aged 50 years or older, the median prevalence of cognitive impairment was 19.0% [19]. Based on data from the Palestinian Central Bureau of Statistics, there were about 205,185 Palestinians aged 50 and older living in the West Bank of Palestine [27]. Using the Raosoft online sample size calculator with a 95% confidence level and a 5% margin of error, we projected a required sample of 237 participants from primary healthcare centers. In our study, 305 participants from these centers were ultimately included. Conversely, in the absence of an official registry for nursing home residents, we contacted all 14 nursing homes in the West Bank and estimated the resident population at 350. Assuming a 50.0% prevalence of cognitive impairment among nursing home residents and using the same sample size parameters, the required sample size was calculated to be 184. However, only 136 nursing home participants met the inclusion criteria and were included in the final analysis. Thus, the total sample size for the study was 441 participants. For primary health care, all participants were selected using convenience sampling after obtaining informed consent. For participants from nursing homes, we were able to sample all eligible residents. Participants were recruited directly by the researcher, who approached them and asked for their age. If their age was above 55 years, they were asked to participate.

### Research tool

Data were collected using a structured questionnaire composed of pre-coded items. Cognitive function was assessed using the Mini-Mental State Examination (MMSE; GTD-USJ), one of the most widely used and validated instruments for this purpose [28,29]. The MMSE(GTD-USJ) comprises six subtests—temporal orientation, spatial orientation, registration, computation and attention, language, and figure copying—yielding a total score ranging from 0 to 30 [29]. This study employed the Arabic version of the A-MMSE(GTD-USJ) —implemented after obtaining the requisite permissions—which has demonstrated sensitivity and specificity rates of 85% and 90%, respectively [29]. In addition, for analytical purposes, MMSE scores were categorized using a cutoff of ≤18 out of 30 to indicate cognitive impairment. The instrument also showed strong reliability, with intra-rater and inter-rater test-retest correlations of 0.89 and 0.72, respectively [29]. The MMSE was administered by a team of three data collectors. The collectors were final-year medical trainees, particularly trained for this project. Their training was conducted under the direct supervision of a consulting neurologist and a neuroscientist to ensure standardization and reliability. The training regimen encompassed a comprehensive examination of the official Arabic version of the MMSE, defined guidelines for administration and scoring, and monitored practice sessions. During the data collection phase, supervisors maintained continuous control and were accessible for consultation to clarify any scoring ambiguities. This organized training, conducted under expert oversight and utilizing a singular, standardized assessment instrument, was established to guarantee a consistent methodology.”

The independent variables collected included sociodemographic and medical characteristics: age (years), gender (male/female), living arrangement (community-dwelling while attending primary healthcare centers or residing in nursing homes), educational level (illiterate, primary school, middle school, high school, or university studies), type of residence (rural, urban, or refugee camp), living arrangement (alone, with family, or with a spouse), number of languages spoken (one or two or more), and body mass index (BMI). Medical history variables encompassed the presence of hypertension, dyslipidemia, diabetes mellitus, coronary heart disease, and heart failure. In addition, information on drug history was collected, including the use of statins, aspirin, metformin, and medications with psychological effects.

### Ethical approval and consent to participate

The research was conducted in compliance with ethical standards and the Declaration of Helsinki, receiving approval from the Institutional Review Board (IRB) of An-Najah National University in Palestine (Ref: Med. Dec. 2023/54). Additionally, consent forms were obtained from all the participants prior to their participation. Confidentiality was preserved by coding the data, not using participants’ names, and limiting data access to the research team.

### Statistical analysis

IBM SPSS was used for statistical analysis. Continuous variables were summarized using medians and interquartile ranges, while categorical variables were expressed as frequencies and percentages. To examine associations between the dependent and independent variables, we employed the Chi-square test/Fisher’s Exact test. The Mann-Whitney U test was used to compare the median age between the two groups based on the MMSE score. A significance level of <0.05 was adopted for all tests.

## Results

### Sociodemographic, medical history, and lifestyle characteristics

Table 1 presents the sociodemographic, medical, and pharmacological characteristics of nursing home residents (*n = 136*). The median age of nursing home residents (136) was 73.0 (61.0–80.8). Most nursing home residents were female (66.9%), and 15.4% resided in the refugee camp. About 41.9% of nursing home participants were illiterate, and 23.5% were smokers. Nearly 58% were overweight or obese. The prevalence of hypertension and diabetes among nursing home residents was 47.8% and 30.9%, respectively. A history of stroke was reported in 14.0% of nursing home residents. In addition, almost 25.7% had Dyslipidemia, 11.8% had coronary artery disease, and 8.1% of nursing home residents had heart failure.

**Table 1:** Sociodemographic, medical, and pharmacological characteristics of nursing home residents (*n = 136*).

Characteristics	Nursing home residents, n (%)
**Age (years), median [IQR]**	73.0 (61.0-80.8)
**Gender (male)**	45 (33.1)
**Smoking status (yes)**	32 (23.5)
**Place of residence**	
City	83 (61.0)
Village	32 (23.5)
Refugee camp	21 (15.4)
**Educational level**	
Illiterate	57 (41.9)
Primary school	32 (23.5)
Middle school	18 (13.2)
High school	16 (11.8)
University	13 (9.6)
**Body Mass Index Categories**	
Underweight	5 (3.7)
Normal weight	52 (38.2)
Overweight	54 (39.7)
Obesity	25 (18.4)
**Spoken languages**	
One	110 (80.9)
Two or more	26 (19.1)
**Medical history**	
Hypertension (yes)	65 (47.8)
Diabetes mellitus (yes)	42 (30.9)
Dyslipidemia (yes)	35 (25.7)
Coronary artery disease (yes)	16 (11.8)
Heart failure (yes)	11 (8.1)
Head trauma (yes)	9 (6.6)
Stroke (yes)	19 (14.0)
**Total**	**136**

IQR: Interquartile range

As shown in Table 2, 45.2% of primary health care attendees were male. The median age of primary health care attendees was 65.0 [60.0–71.0]. Additionally, 27.9% of participants were smokers, and 35.7% resided in rural areas (villages). Regarding participants’ education level, 16.1% were illiterate, and 27.5% had completed university. In reference to comorbidities, 67.9% had hypertension, 59% had Dyslipidemia, and 50.2% had diabetes mellitus. Stroke was present in 6.6% of the attendees, whereas 32.5% had a history of coronary artery disease.

**Table 2:** Sociodemographic, medical history, and lifestyle characteristics of primary health care attendees (n = 305).

Characteristics	Primary health care attendees, n (%)
**Age (years), median [IQR]**	65.0 [60.0, 71.0]
**Gender (male)**	138 (45.2)
**Smoking status (yes)**	85 (27.9)
**Place of residence**	
City	189 (62.0)
Village	109 (35.7)
Refugee camp	7 (2.3)
**Educational level**	
Illiterate	49 (16.1)
Primary school	76 (24.9)
Middle school	40 (13.1)
High school	56 (18.4)
University	84 (27.5)
**Body Mass Index Categories**	
Underweight	2 (0.7)
Normal weight	,39 (12.8)
Overweight	103 (33.8)
Obesity	161 (52.8)
**Spoken languages**	
One	224 (73.4)
Two or more	81 (26.6)
**Medical history**	
Hypertension (yes)	207 (67.9)
Diabetes mellitus (yes)	153 (50.2)
Dyslipidemia (yes)	180 (59.0)
Coronary artery disease (yes)	99 (32.5)
Heart failure (yes)	36 (11.8)
Head trauma (yes)	31 (10.2)
Stroke (yes)	20 (6.6)
**Total**	**305**

IQR: Interquartile range

### Association of cognitive impairment with variables

Table 3 delineates the associations between participant characteristics and cognitive impairment for the nursing home residents. In the nursing home cohort, notable associations with cognitive impairment (MMSE ≤18) were identified for gender (more prevalent in females, p = 0.030), smoking status (more prevalent in non-smokers, p < 0.001), educational attainment (highest among the illiterate, p < 0.001), and monolingualism (p < 0.001). No substantial correlations were identified for the specified medical history comorbidities.

**Table 3:** Associations between cognitive impairment and variables of nursing home residents (n = 136).

	MMSE score		
Characteristic	> 18	≤ 18	p-value^a^
**Age (years), median [IQR]**	68.5 [60.0, 76.0]	75.0 [64.5, 84.0]	**0.021** ^**b**^
**Gender**			
Male, n (%)	23 (16.9)	22 (16.2)	**0.030**
Female, n (%)	29 (21.3)	62 (45.6)	
**Smoking status**			
Yes, n (%)	23 (16.9)	9 (6.6)	**< 0.001**
No, n (%)	29 (21.3)	75 (55.1)	
**Place of residence**			
City, n (%)	38 (27.9)	45 (33.1)	0.051
Village, n (%)	10 (7.4)	22 (16.2)	
Refugee camp, n (%)	4 (2.9)	17 (12.5)	
**Educational level**			
Illiterate, n (%)	5 (3.7)	52 (38.2)	**< 0.001**
Primary school, n (%)	14 (10.3)	18 (13.2)	
Middle school, n (%)	14 (10.3)	4 (2.9)	
High school, n (%)	10 (7.4)	6 (4.4)	
University, n (%)	9 (6.6)	4 (2.9)	
**Body mass index**			
Underweight, n (%)	1 (0.7)	4 (2.9)	0.394
Normal weight, n (%)	19 (14.0)	33 (24.3)	
Overweight, n (%)	19 (14.0)	35 (25.7)	
Obesity, n (%)	13 (9.6)	12 (8.8)	
**Spoken languages**			
One, n (%)	33 (24.3)	77 (56.6)	**< 0.001**
Two or more, n (%)	19 (14.0)	7 (5.1)	
**Medical history**			
**Hypertension**			
No, n (%)	27 (19.9)	44 (32.4)	0.959
Yes, n (%)	25 (18.4)	40 (29.4)	
**Diabetes mellitus**			
No, n (%)	34 (25.0)	60 (44.1)	0.458
Yes, n (%)	18 (13.2)	24 (17.6)	
**Dyslipidemia**			
No, n (%)	40 (29.4)	61 (44.9)	0.577
Yes, n (%)	12 (8.8)	23 (16.9)	
**Coronary artery disease**			
No, n (%)	47 (34.6)	73 (53.7)	0.540
Yes, n (%)	5 (3.7)	11 (8.1)	
**Heart failure**			
No, n (%)	46 (33.8)	79 (58.1)	0.246
Yes, n (%)	6 (4.4)	5 (3.7)	
**Head trauma**			
No, n (%)	48 (35.3)	79 (58.1)	0.692
Yes, n (%)	4 (2.9)	5 (3.7)	
**Stroke**			
No, n (%)	121 (89.0)	15 (11.0)	0.164
Yes, n (%)	42 (30.9)	75 (55.1)	

^a^Chi-square test/Fisher’s Exact test.

^b^Mann-Whitney U Test

IQR: Interquartile range

Table 4 delineates the associations between participant characteristics and cognitive impairment for the primary healthcare attendees. In the cohort of primary health care attendees, significant correlations with cognitive impairment were identified for gender (elevated in females, p < 0.001), smoking status (greater in non-smokers, p < 0.001), educational attainment (highest among the illiterate, p < 0.001), monolingualism (p = 0.003). No substantial correlations were identified for the specified medical history comorbidities.

**Table 4:** Associations between cognitive impairment and variables of primary health care attendees (n = 305).

	MMSE score		
Characteristic	> 18	≤ 18	p-value^a^
**Age (years), median [IQR]**	65.0 [60.0, 70.5]	69.5 [64.0, 74.0]	**0.030** ^b^
**Gender**			
Male, n (%)	137 (44.9)	1 (0.3)	**< 0.001**
Female, n (%)	146 (47.9)	21 (6.9)	
**Smoking status**			
Yes, n (%)	84 (27.5)	1 (0.3)	**0.011**
No, n (%)	199 (65.2)	21 (6.9)	
**Place of residence**			
City, n (%)	173 (56.7)	16 (5.2)	0.352
Village, n (%)	104 (34.1)	5 (1.6)	
Refugee camp, n (%)	6 (2.0)	1 (0.3)	
**Educational level**			
Illiterate, n (%)	31 (10.2)	18 (5.9)	**< 0.001**
Primary school, n (%)	72 (23.6)	4 (1.3)	
Middle school, n (%)	40 (13.1)	0 (0.0)	
High school, n (%)	56 (18.4)	0 (0.0)	
University, n (%)	84 (27.5)	0 (0.0)	
**Body mass index**			
Underweight, n (%)	2 (0.7)	0 (0.0)	0.886
Normal weight, n (%)	36 (11.8)	3 (1.0)	
Overweight, n (%)	97 (31.8)	6 (2.0)	
Obesity, n (%)	148 (48.5)	13 (4.3)	
**Spoken languages**			
One, n (%)	202 (66.2)	22 (7.2)	**0.003**
Two or more, n (%)	81 (26.6)	0 (0.0)	
**Medical history**			
**Hypertension**			
No, n (%)	93 (30.5)	5 (1.6)	0.327
Yes, n (%)	190 (62.3)	17 (5.6)	
**Diabetes mellitus**			
No, n (%)	141 (46.2)	11 (3.6)	0.987
Yes, n (%)	142 (46.6)	11 (3.6)	
**Dyslipidemia**			
No, n (%)	119 (39.0)	6 (2.0)	0.175
Yes, n (%)	164 (53.8)	16 (5.2)	
**Coronary artery disease**			
No, n (%)	190 (62.3)	16 (5.2)	0.590
Yes, n (%)	93 (30.5)	6 (2.0)	
**Heart failure**			
No, n (%)	252 (82.6)	17 (5.6)	0.099
Yes, n (%)	31 (10.2)	5 (1.6)	
**Head trauma**			
No, n (%)	255 (83.6)	19 (6.2)	0.576
Yes, n (%)	28 (9.2)	3 (1.0)	
**Stroke**			
No, n (%)	266 (87.2)	19 (6.2)	0.164
Yes, n (%)	17 (5.6)	3 (1.0)	

^a^Chi-square test/Fisher’s Exact test.

^b^Mann-Whitney U Test

IQR: Interquartile range

## Discussion

Cognitive impairment among older adults is an urgent public health issue with profound implications for individuals, healthcare providers, and society as a whole. This study is the first of its kind within the Palestinian healthcare context, describing cognitive function in nursing home residents and community-dwelling older adults, with each cohort presented as an independent clinical profile.

Among nursing home residents, 61.8% met the threshold for major cognitive impairment (Mini-Mental State Examination [MMSE] ≤18), Higher than the 74.3% reported in comparable nursing home studies in the region [30]. While the higher prevalence of cognitive impairment is expected in institutionalized populations [22], the more clinically distinctive finding is the low mean age of residents (72.1 years). This is younger than the reported ages of nursing home residents in multiple studies worldwide [20,31,32]. Given that nursing home culture remains underdeveloped in the Arab world [33], this early institutionalization most plausibly reflects structural deficits in community-based care rather than an accelerated aging trajectory. The near-absence of home-care alternatives forces Palestinian older adults without significant family support into residential placement. Socioeconomic hardship further compounds this dynamic [34,35].

Among the 305 primary healthcare attendees, the prevalence of cognitive impairment was 7.2%, substantially lower than in the nursing home cohort and consistent with the expectation that community-dwelling older adults represent a less severely impaired population [19], as well as with estimates from neighboring Arab countries [36,37].

The identification of specific factors associated with cognitive decline further illuminates actionable pathways for intervention. Lower educational levels and monolingualism emerged as potent risk factors, underscoring the potential benefits of lifelong learning and bilingual education in bolstering cognitive reserve [38]. Similarly, hypertension and dyslipidemia were found to be significant predictors of cognitive deterioration, suggesting that rigorous cardiovascular and metabolic control could substantially reduce cognitive risks. In the case of Diabetes, The most recent consensus estimates the prevalence of Diabetes in the west bank in adults between 40–69 to be 23.3% [39], suggesting that the reported prevalence in our study is above the national average, this is most likely to be caused by the sample acquisition from primary healthcare centers, whose attendees will be more likely to suffer from chronic diseases such as diabetes, than the general population. Diabetes was found to be significantly correlated with cognitive impairment in most research [40]. However, a study of 100,000 subjects found only modest, non-statistically significant increases [41]. The notably high prevalence of diabetes in nursing homes highlights the necessity for targeted public health interventions and for specialists with expertise in diabetes management within this population.

Smokers showed a lower prevalence of MMSE ≤18 than non-smokers. This finding should not be construed as evidence of a protective effect of tobacco on cognition; the established biological links between smoking, cerebrovascular pathology, and neurodegeneration are well-documented [42]. The most plausible explanation is survivor bias: individuals who smoke and develop significant cognitive impairment are disproportionately likely to die before reaching institutionalization, leaving a surviving cohort that is biologically selected. This phenomenon has been previously described in the Italian Longitudinal Study on Aging [41].

These findings offer healthcare providers and policymakers a local prevalence baseline that may inform future planning to modify health policies to include comprehensive risk screening, early cognitive assessment, and integrated intervention programs tailored to the cultural and socioeconomic context of Palestine. Cognitive stimulation therapy has demonstrated benefit for cognitive function in older adults across care settings [43].

The higher prevalence of major cognitive impairment observed in nursing homes in this study, combined with the more structured living environment they provide, suggests that this setting may be a priority context for evaluating and piloting such programs. Longitudinal research is needed to establish the efficacy and feasibility of targeted interventions within the Palestinian institutional context before broader implementation can be recommended.

## Strength of the study

This study possesses several strengths that reinforce the validity of its findings. First, its novelty as the first comprehensive descriptive study in Palestine fills an important gap in the literature. Second, the inclusion of both institutionalized and community-dwelling older adults allows for a broad perspective on cognitive health across different care settings. Third, the relatively high participation rate among nursing home residents (136 of 350, 38.8%) enhances the representativeness of the findings for this population. Finally, the use of the Arabic version of the Mini-Mental State Examination A-MMSE(GTD-USJ)—an instrument recognized for its reliability and validity—provides robust measures of cognitive function.

## Limitations of the study

Despite these strengths, several limitations must also be acknowledged. The cross‐sectional design limits our ability to draw causal inferences about the relationship between risk factors and cognitive decline. The reliance on convenience sampling, particularly within the primary healthcare centers, may have introduced selection bias and limited the generalizability of the findings to all older Palestinian adults. Additionally, excluding older adults who do not regularly attend healthcare centers may omit a subgroup that may exhibit distinct cognitive profiles. Finally, the removal of incomplete data responses might have led to an underrepresentation of more severely impaired individuals, further impacting the study’s findings.

## Conclusion

This descriptive study documents the high prevalence of major cognitive impairment among nursing home residents in the West Bank, occurring in a cohort substantially younger than comparable populations worldwide. Illiteracy, refugee origin, monolingualism, and female predominance characterize the most cognitively vulnerable residents, reflecting possible structural and cognitive inequities that predate institutionalization. Among primary healthcare attendees, dyslipidemia and stroke history were the principal vascular correlates of cognitive impairment. These findings establish the first baseline prevalence data for both care settings in Palestine and underscore the need for geriatric care standards tailored to the local population. Given the cross-sectional design, no causal claims can be made. The data support investment in longitudinal cohort research to characterize the natural history of cognitive decline in this population, as well as enhanced management of modifiable risk factors and evidence-based cognitive stimulation programs within nursing facilities.

## Supporting information

S1 Data All data (1). (XLSX)
